# TAK1 Mediates ROS Generation Triggered by the Specific Cephalosporins through Noncanonical Mechanisms

**DOI:** 10.3390/ijms21249497

**Published:** 2020-12-14

**Authors:** Midori Suzuki, Yukino Asai, Tomohiro Kagi, Takuya Noguchi, Mayuka Yamada, Yusuke Hirata, Atsushi Matsuzawa

**Affiliations:** Laboratory of Health Chemistry, Graduate School of Pharmaceutical Sciences, Tohoku University, 6-3 Aoba, Aramaki, Aoba-ku, Sendai 980-8578, Japan; midori.suzuki.r5@dc.tohoku.ac.jp (M.S.); yukino.asai.r4@dc.tohoku.ac.jp (Y.A.); toho131@dc.tohoku.ac.jp (T.K.); mayuka.yamada.p8@dc.tohoku.ac.jp (M.Y.); yusuke.hirata.d8@tohoku.ac.jp (Y.H.)

**Keywords:** transforming growth factor-β (TGF-β)-activated kinase 1 (TAK1), reactive oxygen species (ROS), antibacterial agents, macrophages

## Abstract

It is known that a wide variety of antibacterial agents stimulate generation of reactive oxygen species (ROS) in mammalian cells. However, its mechanisms are largely unknown. In this study, we unexpectedly found that transforming growth factor-β (TGF-β)-activated kinase 1 (TAK1) is involved in the generation of mitochondrial ROS (mtROS) initiated by cefotaxime (CTX), one of specific antibacterial cephalosporins that can trigger oxidative stress-induced cell death. TAK1-deficient macrophages were found to be sensitive to oxidative stress-induced cell death stimulated by H_2_O_2_. Curiously, however, TAK1-deficient macrophages exhibited strong resistance to oxidative stress-induced cell death stimulated by CTX. Microscopic analysis revealed that CTX-induced ROS generation was overridden by knockout or inhibition of TAK1, suggesting that the kinase activity of TAK1 is required for CTX-induced ROS generation. Interestingly, pharmacological blockade of the TAK1 downstream pathways, such as nuclear factor-κB (NF-κB) and mitogen-activated protein kinase (MAPK) pathways, did not affect the CTX-induced ROS generation. In addition, we observed that CTX promotes translocation of TAK1 to mitochondria. Together, these observations suggest that mitochondrial TAK1 mediates the CTX-induced mtROS generation through noncanonical mechanisms. Thus, our data demonstrate a novel and atypical function of TAK1 that mediates mtROS generation triggered by the specific cephalosporins.

## 1. Introduction

Transforming growth factor-β (TGF-β)-activated kinase 1 (TAK1, also known as MAP3K7) is a member of the mitogen-activated protein (MAP) kinase kinase kinase (MAP3K) family [1,2]. TAK1 preferentially activates both the mitogen-activated protein kinases (MAPKs) pathways, such as the c-Jun N-terminal kinase (JNK) and p38 MAPK pathways, and nuclear factor-κB (NF-κB) signaling pathways, and thereby induces a wide variety of cellular responses [1]. A series of studies to characterize the function of TAK1 have revealed that TAK1 is activated by proinflammatory mediators including tumor necrosis factor (TNF)-α, interleukin (IL)-1β, Toll-like receptor (TLR) ligands, and functions as an essential component in inflammatory signaling [2,3]. In addition, exogenous or environmental stresses, such as DNA-damaging agents, UV irradiation, and osmotic shock, also activate TAK1 [4,5,6]. Furthermore, recent evidence has demonstrated the importance of TAK1 in oxidative stress responses [7,8,9,10]. Thus, TAK1 is conceived as a multifunctional kinase that can respond to a wide range of stimuli. Although knockout (KO) of TAK1 leads to embryonic lethality due to developmental defects in mice, TAK1-deficient cells, including mouse embryonic fibroblasts (MEF), B cells, T cells, and keratinocytes, are viable [11,12]. On the other hand, TAK1-deficient macrophages spontaneously die, suggesting that TAK1 plays an essential role in survival of macrophages [13,14]. However, the biological functions of TAK1 in macrophages are not fully understood.

Reactive oxygen species (ROS) are continuously produced by aerobic metabolism. Since excessive ROS accumulation elicits oxidative stress that causes severe cellular dysfunctions, cells choose to eliminate ROS by upregulating various antioxidant proteins or to induce cell death [15]. TAK1 contributes to cell survival by promoting the antioxidative defence responses through the NF-E2-related factor 2 (Nrf2) pathways, although TAK1 can enhance cell death under some conditions [1,16,17,18]. On the other hand, apoptosis signal-regulating kinase 1 (ASK1), another member of MAP3K family, induces apoptosis by activating the JNK and p38 MAPK signaling pathways [19,20,21]. Hence, the life and death decisions under oxidative stress conditions seem to be tightly regulated by TAK1 and ASK1.

Antibacterial cephalosporins, a class of β-lactam antibiotics that contain β-lactam and hetero six-membered rings as basic skeletons, are broad-spectrum antibiotics used to control various infectious diseases. Cefotaxime (CTX), a third-generation cephalosporin, is one of the most commonly prescribed antibiotics for the treatment of infectious diseases [22]. Recent evidence has shown that the bactericidal antibiotics cause mitochondrial dysfunction and oxidative stress in mammalian cells [23,24]. Indeed, we have recently demonstrated that some of cephalosporins including CTX has an ability to stimulate mitochondrial reactive oxygen species (mtROS) generation in human cells [25,26]. However, the mechanisms underlying the generation of mtROS initiated by these cephalosporins remain unclear.

In this study, we investigated the biological roles of TAK1 in oxidative stress responses induced by accumulation of CTX-driven mtROS, and unexpectedly found that TAK1 is involved in the mtROS generation induced by CTX in macrophages, which is a novel function of TAK1. More interestingly, typical downstream of TAK1 does not appear to be involved in the mtROS generation, suggesting that TAK1 mediates CTX-induced ROS generation through noncanonical mechanisms.

## 2. Results

### 2.1. TAK1 Is Required for Oxidative Stress-Induced Cell Death Stimulated by CTX

**Figure 1:**
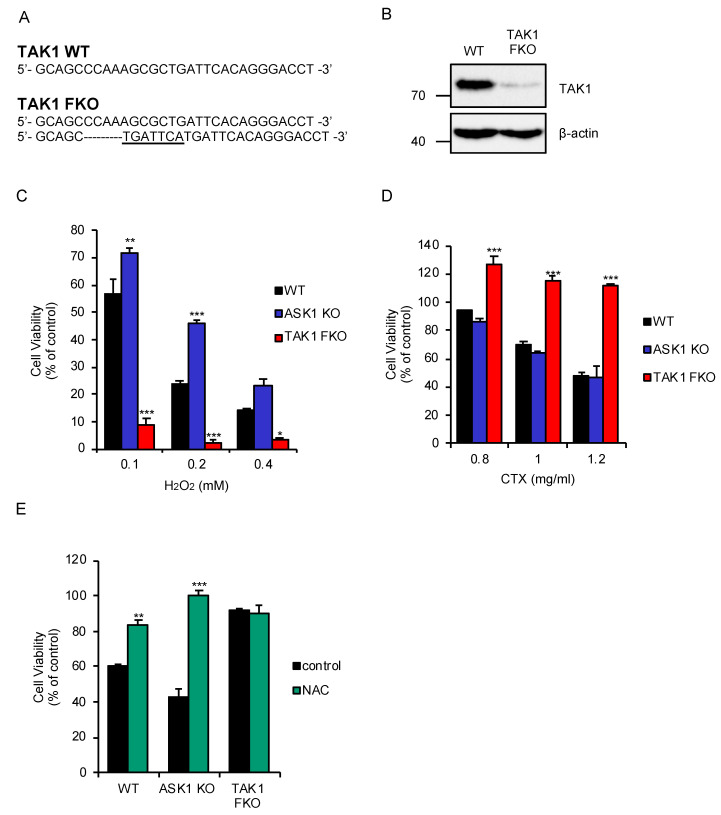
Transforming growth factor-β (TGF-β)-activated kinase 1 (TAK1) is required for oxidative stress-induced cell death stimulated by cefotaxime (CTX). (**A**) DNA sequences around the guide RNAs (gRNAs) target sites of TAK1. (**B**) Immunoblot analysis of TAK1 in RAW264.7 cells. RAW264.7 cells were subjected to immunoblotting with the indicated antibodies. β-actin was used as a loading control. (**C**) TAK1 functional knockout (FKO) RAW264.7 cells were sensitive to H_2_O_2_-induced cell death. RAW264.7 cells were treated with the indicated concentrations of H_2_O_2_ for 3.5 h, and then subjected to cell viability assay. (**D**) TAK1 FKO RAW264.7 cells were resistant to CTX-induced cell death.** RAW264.7 cells were treated with 1 mg/mL cefotaxime for 48 h, and then subjected to cell viability assay. (**E**) The effect of N-acetylcysteine (NAC) on CTX-induced cell death in macrophages.** RAW264.7 cells were treated with 1 mg/mL cefotaxime for 48 h in the presence of the antioxidant NAC (1 mM), and then subjected to cell viability assay. (**C**–**E**) Data shown are the mean ± SD (*n* = 3). Significant differences were assessed by one-way ANOVA, followed by Tukey–Kramer test; * *p* < 0.05, ** *p* < 0.01, *** *p* < 0.001 (versus control). All data are representative of at least three independent experiments.

We succeeded in establishing a clone of TAK1 functional knockout (FKO) cells in the murine macrophage-like RAW264.7 cells, yet primary macrophages spontaneously die when TAK1 is knocked out. Although the clone, generated by using the CRISPR/Cas9 system, is still harboring a TAK1 wild-type (WT) allele (Figure 1A), immunoblot analysis revealed that the protein expression of TAK1 is almost abolished (Figure 1B). We therefore regarded the TAK1-deficient RAW264.7 cells as FKO cells, and then investigated the potential role of TAK1 in oxidative stress response in macrophages. We firstly found that TAK1 FKO RAW264.7 cells were sensitive to oxidative stress-induced cell death stimulated by H_2_O_2_, whereas ASK1 KO RAW264.7 cells were significantly resistant, as previously demonstrated (Figure 1C) [19,27]. We next examined whether similar results would be obtained with CTX, which has been found to generate mtROS [25,26]. Curiously, however, TAK1 FKO RAW264.7 cells exhibited strong resistance, and ASK1 KO RAW264.7 cells did not show resistance to CTX-induced cell death (Figure 1D). To exclude the possibility that CTX does not initiate oxidative stress-induced cell death in macrophages, we tested whether the antioxidant such as *N*-acetylcysteine (NAC) can rescue CTX-induced cell death in macrophages. As shown in Figure 1E, co-treatment with NAC clearly inhibited CTX-induced cell death in WT and ASK1 KO RAW264.7 cells. Together, these observations suggest that TAK1 but not ASK1 is required for oxidative stress-induced cell death stimulated by CTX in macrophages.

### 2.2. TAK1 Is Required for CTX-Induced ROS Generation

**Figure 2:**
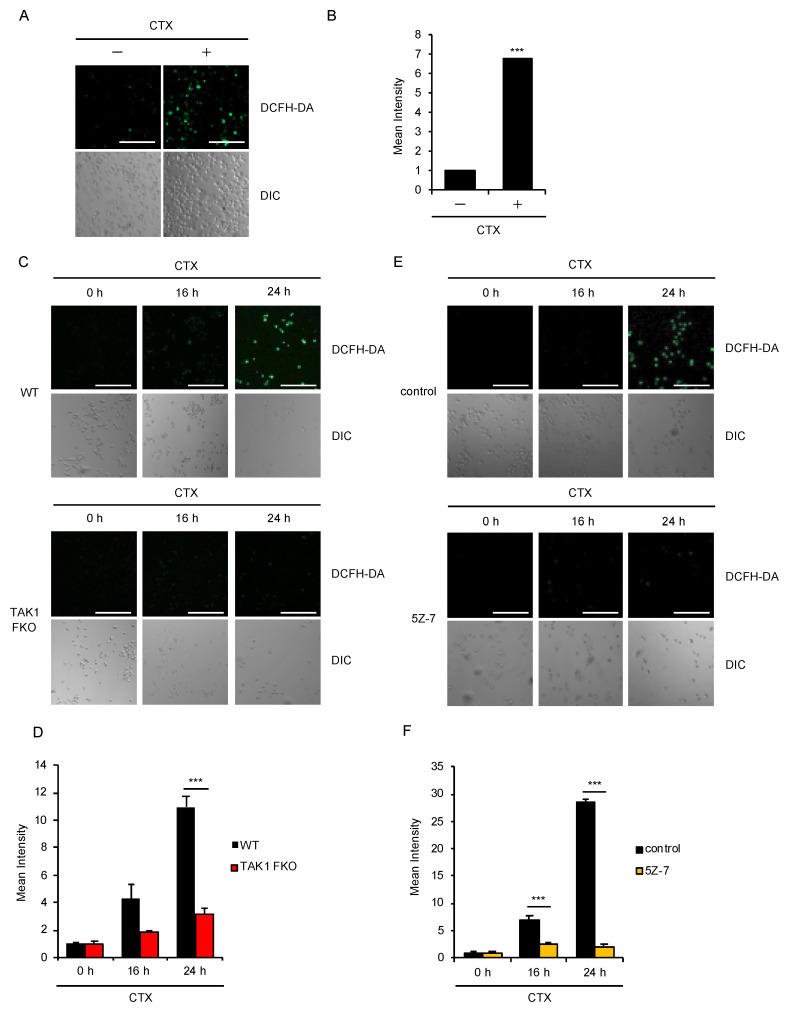
TAK1 is required for CTX-induced ROS generation. (**A**,**B**) CTX-induced ROS generation in macrophages. Bone marrow derived macrophages (BMDMs) were treated with 1 mg/mL cefotaxime for 24 h, and then treated with 10 μM 2′,7′-dichlorodihydrofluorescein diacetate (DCFH-DA). Fluorescence images (**A**) and intensity (**B**) of BMDMs were acquired as described in the materials and methods section. Cell morphology was determined by Nomarski differential interference contrast (DIC) microscopy (Scale bar, 200 μm). (**C**,**D**) Requirement of TAK1 for CTX-induced ROS generation in macrophages. RAW264.7 cells were treated with 0.8 mg/mL cefotaxime for 16 h and 24 h, and then treated with 10 μM DCFH-DA. Fluorescence images (**C**) and intensity (**D**) of RAW264.7 cells were acquired as described in the materials and methods section. Cell morphology was determined by DIC microscopy (Scale bar, 200 μm). (**E**,**F**) Requirement of the kinase activity of TAK1 for CTX-induced ROS generation in macrophages. RAW264.7 cells were treated with 1 mg/mL cefotaxime for 16 h and 24 h in the presence of the TAK1 inhibitor 5Z-7 (800 nM) and then treated with 10 μM DCFH-DA. Fluorescence images (**E**) and intensity (**F**) of RAW264.7 cells were acquired as described in the materials and methods section. Cell morphology was determined by DIC microscopy (Scale bar, 200 μm). (**B**,**D**,**F**) Data shown are the mean ± SD (*n* = 3). Significant differences were assessed by one-way ANOVA, followed by Tukey–Kramer test; *** *p* < 0.001 (versus control). All data are representative of at least three independent experiments.

We therefore investigated how TAK1 promotes CTX-induced cell death. Microscopic analysis using the ROS indicator 2′,7′-dichlorodihydrofluorescein diacetate (DCFH-DA) revealed that CTX actually induces ROS generation in bone marrow derived macrophages (BMDMs) (Figure 2A,B). Therefore, we next examined the ROS generation in TAK1 FKO RAW264.7 cells. Interestingly, CTX-induced ROS generation was strongly attenuated in TAK1 FKO RAW264.7 cells, when compared with WT RAW264.7 cells (Figure 2C,D). Moreover, the TAK1 kinase inhibitor 5Z-7-oxozeaenol (5Z-7) also significantly inhibited CTX-induced ROS generation in WT RAW264.7 cells (Figure 2E,F). Thus, these results suggest that the kinase activity of TAK1 is required for CTX-induced ROS generation in RAW264.7 cells.

### 2.3. Activation of the MAPK and NF-κB Pathways Are not Required for CTX-Induced ROS Generation

**Figure 3:**
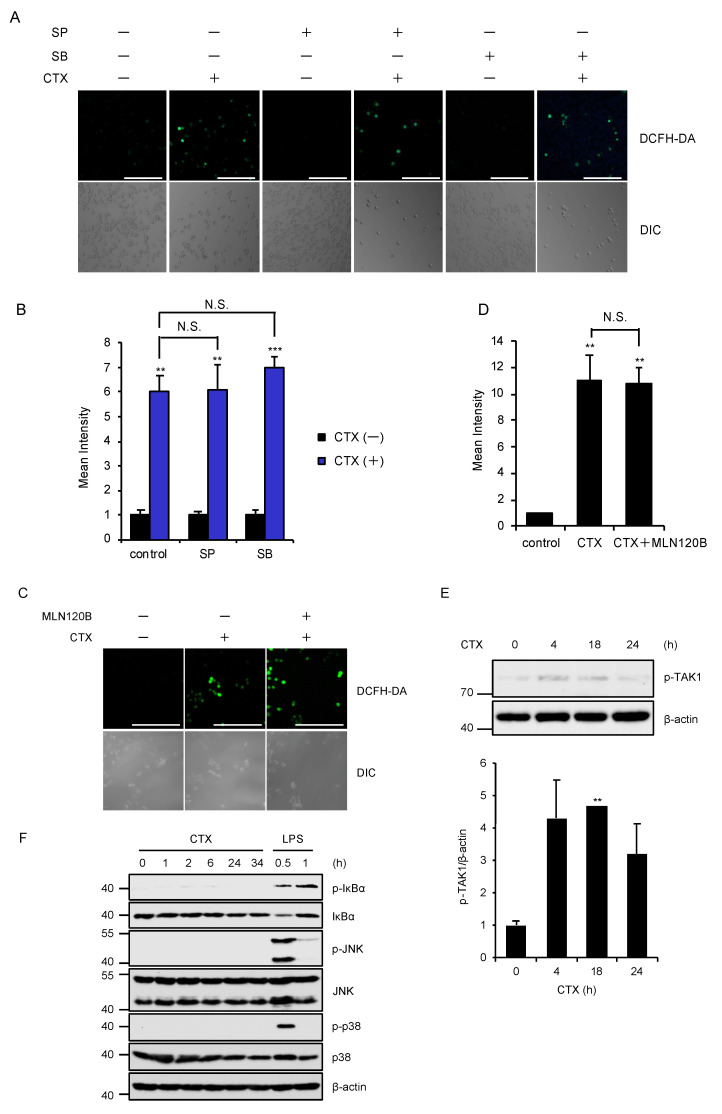
Activation of the MAPK and NF-κB pathways are not required for CTX-induced ROS generation. (**A**,**B**) The effect of specific inhibitors for JNK and p38 MAPK signaling pathways on CTX-induced ROS generation in macrophages. RAW264.7 cells were treated with 0.8 mg/mL cefotaxime for 24 h in the presence of the p38 inhibitor SB203580 (SB, 5 μM), and the JNK inhibitor SP600125 (SP, 5 μM), and then treated with 10 μM DCFH-DA. Fluorescence images (**A**) and intensity (**B**) of RAW264.7 cells were acquired as described in the materials and methods section. Cell morphology was determined by Nomarski differential interference contrast (DIC) microscopy (Scale bar, 200 μm). (**C**,**D**) The effect of a specific inhibitor for IκB kinases (IKKs) (MLN120B) on CTX-induced ROS generation in macrophages. RAW264.7 cells were treated with 0.8 mg/mL cefotaxime for 24 h in the presence of the IKK inhibitor MLN120B (5 μM), and then treated with 10 μM DCFH-DA. Fluorescence images (**C**) and intensity (**D**) of RAW264.7 cells were acquired as described in the materials and methods section. Cell morphology was determined by Nomarski differential interference contrast (DIC) microscopy. (Scale bar, 200 μm). (**E**) CTX-induced TAK1 activation. RAW264.7 cells were treated with 0.8 mg/mL cefotaxime for the indicated periods. Cell lysates were subjected to immunoblotting with the indicated antibodies, and the relative expression of phospho-TAK1 was quantified using Image Lab software from Bio-Rad. β-actin was used as a loading control. The data is a representative of three independent experiments, and graphs depict the value of means and S.D. of three independent experiments. Significant differences were determined by Student’s *t*-test; ** *p* < 0.01. (**F**) CTX-induced NF-κB and MAPK activation. RAW264.7 cells were treated with 0.6 mg/mL cefotaxime or 100 ng/mL LPS for the indicated periods. Cell lysates were subjected to immunoblotting with the indicated antibodies. β-actin was used as a loading control. (**B**,**D**) Data shown are the mean ± SD (*n* = 3). Significant differences were assessed by one-way ANOVA, followed by Tukey–Kramer test; *** *p* < 0.001, ** *p* < 0.01. N.S.: Not significant. All data are representative of at least three independent experiments.

We next examined the involvement of the canonical downstream pathways of TAK1 in CTX-induced ROS generation. However, pharmacological blockade of the MAPK pathways using specific inhibitors for JNK and p38 MAPK signaling pathways did not affect CTX-induced ROS generation in RAW264.7 cells (Figure 3A,B). Moreover, the inhibitor of IκB kinases (IKKs), which govern the activation of NF-κB signaling pathways, also failed to inhibit CTX-induced ROS generation (Figure 3C,D). These findings suggest that TAK1 mediates CTX-induced ROS generation without activating the MAPK and NF-κB signaling pathways. Indeed, CTX slightly but certainly promotes the phosphorylation of TAK1 at the active site (Thr184/187) (Figure 3E). However, CTX failed to enhance the phosphorylation levels of IκBα (an indicator of the NF-κB activation), JNK and p38 MAPK, whereas lipopolysaccharide (LPS) clearly enhanced (Figure 3F). Collectively, these findings raise the possibility that TAK1 mediates CTX-induced ROS generation by activating alternative pathways rather than the MAPK and NF-κB signaling pathways.

### 2.4. CTX-Driven Mitochondrial Damage Is Mitigated by Loss of TAK1

**Figure 4:**
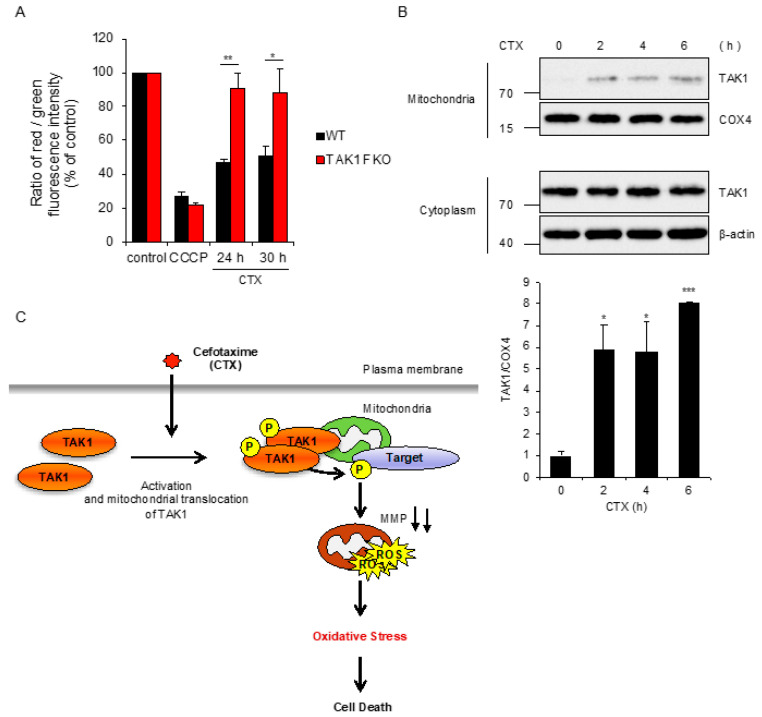
CTX-driven mitochondrial damage is mitigated by loss of TAK1. (**A**) RAW264.7 cells were treated with CCCP 10 μM for 6 h or 0.8 mg/mL cefotaxime for the indicated periods, and then treated with 2 μM JC-1. Data shown are the mean ± SD (*n* = 3). Significant differences were assessed by one-way ANOVA, followed by Tukey–Kramer test; ** *p* < 0.01, * *p* < 0.05. (**B**) RAW264.7 cells were treated with 0.8 mg/mL cefotaxime for the indicated periods, and then the cytoplasmic and mitochondrial fractions were subjected to immunoblotting with the indicated antibodies, and relative expression of TAK1 in mitochondrial fraction was quantified using Image Lab software from Bio-Rad. β-actin and COX4 were used as a loading control of cytoplasmic and mitochondrial fractions, respectively. The data is a representative of three independent experiments, and graphs depict the value of means and S.D. of three independent experiments. Significant differences were determined by Student’s *t*-test; *** *p* < 0.001, * *p* < 0.05. (**C**) A schematic model to explain our study was described. Our findings suggest that mitochondrial TAK1 phosphorylates unknown target and thereby induces the collapse of MMP accompanied with mtROS generation, leading to oxidative stress-induced cell death, in the presence of CTX.

We next explored the mechanisms by which TAK1 mediates CTX-induced ROS generation. In regard to mtROS generation, it is known that collapse of mitochondrial membrane potential (MMP) allows leakage of ROS from mitochondria [28]. We thus examined whether TAK1 is involved in the collapse of MMP induced by CTX, using the fluorescent probe 5,5′,6,6′-tetrachloro-1,1′,3,3′-tetraethylbenzimidazolyl-carbocyanine iodide (JC-1), as a MMP indicator [29]. As shown in Figure 4A, carbonyl cyanide m-chlorophenylhydrazine (CCCP), a representative uncoupling agent, clearly reduced MMP in both WT and TAK1 FKO RAW264.7 cells, and CTX also reduced MMP in WT RAW264.7 cells. However, CTX failed to do so in TAK1 FKO RAW264.7 cells, suggesting that TAK1 is required for CTX-induced collapse of MMP. Moreover, subcellular fractionation analysis revealed that TAK1 is enriched in mitochondrial fractions in a CTX stimulation-dependent manner (Figure 4B). Together, these results suggest that CTX promotes the mitochondrial translocation of TAK1, and then mitochondrial TAK1 mediates the collapse of MMP, which may be responsible for CTX-induced ROS generation.

## 3. Discussion

The great contribution of antibiotics to human health is indisputable. However, little attention has been paid to host cellular processes initiated by antibiotics, which may be responsible for the adverse reactions to antibiotics. Accumulating evidence suggests that the bactericidal antibiotics cause oxidative stress through mtROS generation in mammalian cells [23,24]. To elucidate the mechanisms by which antibiotics initiate oxidative stress may provide therapeutic benefits. However, its mechanisms remain unknown. In the present study, we unexpectedly found that TAK1 mediates CTX-induced ROS generation, to our knowledge, which is the first report of a key component that mediates ROS generation triggered by antibiotics. TAK1 is widely known as a stress-responsive kinase that activates the JNK and p38 MAPK but not ERK pathways [30]. In addition, TAK1 also activates the NF-κB signaling pathways [1]. However, interestingly, our results show that these typical downstream pathways of TAK1 are not involved in TAK1-mediated ROS generation. Since CTX drives the mitochondrial translocation of TAK1, phosphorylation targets of TAK1 may exist in mitochondria or cytoplasm surrounding mitochondria. Moreover, our results that TAK1 mediates CTX-induced reduction of MMP imply that phosphorylation targets of TAK1 are involved in the regulation of mitochondrial membrane permeability. The antiapoptotic B-cell lymphoma (BCL) 2 family proteins are proposed as an example of the possible candidates. In particular, it has been reported that B-cell lymphoma-2-associated X (BAX) protein is activated by phosphorylation and thereby increases mitochondrial membrane permeability [31]. Therefore, this finding raises the possibility that TAK1 targets BAX or its related proteins. In any case, considering that TAK1 is involved in the regulation of mitochondrial biogenesis and function [32,33], it is formally possible that TAK1 physiologically regulates the membrane permeability of mitochondria.

The unexpected function of TAK1 that induces cell death by promoting mtROS generation is inconsistent with the already-known functions of TAK1 that contribute to cellular survival. However, there are cases in which ROS generation contributes to cellular survival. For instance, some oxidants activate epidermal growth factor receptor (EGFR) signaling, a representative survival pathway, through the inactivation of its negative regulators such as protein phosphatases by S-oxidation, raising the possibility that ROS are intentionally generated in order to activate the survival signaling pathway [34]. Therefore, TAK1 may physiologically translocate to mitochondria, and thereby promotes mtROS generation, leading to activation of the survival signaling pathways including the Nrf2 pathway. However, it is likely that CTX perturbs the TAK1 function, and then undergoes excessive ROS accumulation up to cellular lethal levels. Interestingly, our recent studies have shown that elaidic acid, a trans-fatty acid (TFA) most abundantly included in processed foods, promotes oxidative stress-induced apoptosis by causing hyperactivation of ASK1, which provides insight into the mechanisms underlying the pathogenesis of TFA-induced atherosclerosis [27,35]. These examples demonstrate that the pharmaceutical agents such as CTX and food additives can adversely affect oxidative stress responses by perturbations in cell signaling. Elucidation of the cell signaling perturbations initiated by various chemical substances may make it possible to understand the mechanisms underlying the pathogenesis of oxidative stress-related diseases. Thus, although further research is needed to uncover the precise mechanims, TAK1 may be a notable molecular target of CTX, which provides insights into how the cephalosporins cause the severe side effects such as anaphylaxis, acute kidney injury, and toxic epidermal necrolysis.

## 4. Materials and Methods

### 4.1. Cell Culture and Reagents

RAW264.7 cells obtained from ATCC were grown in RPMI 1640, 10% heat-inactivated fetal bovine serum (FBS), and 1% penicillin–streptomycin solution, at 37 °C under a 5% CO_2_ atmosphere. BMDMs were isolated as described previously [19], and were cultured in RPMI 1640 containing 20% medium conditioned by L929 mouse fibroblasts and 10% heat-inactivated fetal bovine serum, and 1% penicillin–streptomycin solution in 5% CO_2_ at 37 °C atmosphere. Cefotaxime Sodium Salt, SP600125, NAC, and H_2_O_2_ were purchased from Wako (Tokyo, Japan). TAK1 inhibitor 5Z-7 and SB203580 were purchased from Santa Cruz (Dallas, TX, USA). IKK inhibitor MLN120B, and CCCP were purchased from Merck Millipore (Burlington, VT, USA). The antibodies used were against TAK1, p38 MAPK, JNK, phospho-p38 MAPK, and phospho-JNK (Cell Signaling, Danvers, MA, USA), phospho-TAK1 (Thermo Fisher Scientific, Hampton, NH, USA), COX4 (Santa Cruz), and β-actin (Wako).

### 4.2. Colorimetric Cell Viability Assay

Cell viability assay was performed as described previously [36]. Cells were seeded on 96-well plates. After indicated stimulation, cell viability was determined using Cell Titer 96 Cell Proliferation Assay (Promega, Madison, WI, USA), according to the manufacturer’s protocol. The absorbance was read at 492 nm using a microplate reader. Data are normalized to control (100%) without stimulus.

### 4.3. Immunoblot Analysis

Cells were lysed with the 1% Triton X-100 buffer [20 mM Tris-HCl (pH 7.4), 150 mM NaCl, 1% Triton-X100, 10% Glycerol, and 1% protease inhibitor cocktails (Nacalai Tesque, Kyoto, Japan)]. After centrifugation, the cell extracts were resolved by SDS-PAGE and analyzed as described previously [37]. The blots were developed with ECL (Merck Millipore).

### 4.4. Generation of Knockout Cell Lines

*ASK1* knockout RAW 264.7 cells were generated and characterized in the previous study [27]. *TAK1* knockout RAW 264.7 cells were generated using the CRISPR/Cas9 system as described previously [38]. Guide RNAs (gRNAs) were designed to target a region in the exon 5 of *ASK1* gene (5′-GGTATGGATTCCCGGAAGTA-3′), and the exon 5 of *TAK1* gene (5′-TGTGGAAAGGACGAAACACCGGATCGACTACAAGGAGATCG-3′) using CRISPRdirect. gRNA-encoding oligonucleotide was cloned into lentiCRISPRv2 plasmid (Addgene, Watertown, MA, USA), and knockout cells were established as previously described [39]. To determine the mutations of *ASK1* and *TAK1* in cloned cells, genomic sequence around the target region was analyzed by PCR-direct sequencing using extracted DNA from each clone as a template and the following primers: 5′-GTCATGCGTTTTCCTC-3′ and 5′-ATATTGTCTACCCGTTGC-3′ for *ASK1*, 5′-ATCATGTCGACAGCCTCCGC-3′ and 5′-TCCTGGACTCTAACACCACT-3′ for *TAK1*.

### 4.5. Bioimaging and Quantification of ROS

ROS detection assay was performed as described previously [40]. RAW264.7 cells or BMDMs were seeded on glass plates. After stimulation, cells were treated with 10 µM DCFH-DA for 30 min at 37  °C. After washing with phosphate-buffered saline (PBS), the intracellular ROS generation was observed using a Zeiss LSM800 laser confocal microscope (Carl Zeiss, Oberkochen, Germany) or Olympus Fluoview FV1000 laser confocal microscope (Olympus, Tokyo, Japan), and the images were processed with Zen software or FV10-ASW software. The fluorescence images were obtained from three deferent fields of view. Data shown are the mean ± SD of three images.

### 4.6. Isolation of Mitochondrial Fraction

Mitochondria were isolated as described previously [41]. Cells were washed twice with ice-cold PBS and were lysed by using a glass Dounce homogenizer in homogenization buffer [20 mM Hepes (pH 7.9), 0.22 M mannitol, 0.08 M sucrose, and 1% protease inhibitor cocktails (Nacalai Tesque)]. The homogenate was centrifuged at 310× *g* for 2 min at 4 °C to remove debris including nucleus. The supernatant was recentrifuged at 5000× *g* for 5 min at 4 °C. The supernatant was used as cytoplasm fractions, and precipitate was used as mitochondria enriched fractions.

### 4.7. Mitochondrial Membrane Potential Assay

Mitochondrial membrane potential was measured by JC-1 MitoMP Detection Kit (MT09) (Dojindo, Kumamoto, Japan) according to the manufacturer’s instructions. JC-1 dye is captured by mitochondria and exhibits strong red fluorescence. However, loss of mitochondrial membrane potential blocks the capture, and then JC-1 exhibits green fluorescence. Thus, reduction of mitochondrial membrane potential can be measured by the ratio of the red/green fluorescence [29].
